# Development Real-Time PCR Assays to Genetically Differentiate Vaccinated Pigs From Infected Pigs With the Eurasian Strain of African Swine Fever Virus

**DOI:** 10.3389/fvets.2021.768869

**Published:** 2021-10-27

**Authors:** Lauro Velazquez-Salinas, Elizabeth Ramirez-Medina, Ayushi Rai, Sarah Pruitt, Elizabeth A. Vuono, Nallely Espinoza, Douglas P. Gladue, Manuel V. Borca

**Affiliations:** ^1^Agricultural Research Service, United States Department of Agriculture, Plum Island Animal Disease Center, Greenport, NY, United States; ^2^Department of Anatomy and Physiology, Kansas State University, Manhattan, KS, United States; ^3^Oak Ridge Institute for Science and Education (ORISE), Oak Ridge, TN, United States; ^4^Department of Pathobiology and Population Medicine, Mississippi State University, Mississippi, MS, United States

**Keywords:** ASFV, ASF real time PCR, genetic DIVA test, live attenuated vaccine, phylogenetics

## Abstract

Currently, African swine fever virus (ASFV) represents one of the most important economic threats for the global pork industry. Recently, significant advances have been made in the development of potential vaccine candidates to protect pigs against this virus. We have previously developed attenuated vaccine candidates by deleting critical viral genes associated with virulence. Here, we present the development of the accompanying genetic tests to discriminate between infected and vaccinated animals (DIVA), a necessity during an ASFV vaccination campaign. We describe here the development of three independent real-time polymerase chain reaction (qPCR) assays that detect the presence of MGF-360-12L, UK, and I177L genes, which were previously deleted from the highly virulent Georgia strain of ASFV to produce the three recombinant live attenuated vaccine candidates. When compared with the diagnostic reference qPCR that detects the p72 gene, all assays demonstrated comparable levels of sensitivity, specificity, and efficiency of amplification to detect presence/absence of the ASFV Georgia 2007/1 strain (prototype virus of the Eurasian lineage) from a panel of blood samples from naïve, vaccinated, and infected pigs. Collectively, the results of this study demonstrate the potential of these real-time PCR assays to be used as genetic DIVA tests, supporting vaccination campaigns associated with the use of ASFV-ΔMGF, ASFV-G-Δ9GL/ΔUK, and ASFV-ΔI177L or cell culture adapted ASFV-ΔI177LΔLVR live attenuated vaccines in the field.

## Introduction

African swine fever virus (ASFV), an arbovirus, and unique member of the *Asfarviridae* family, is a double-stranded DNA virus with a varying genome length that ranges between 170 and 193 kbp, encoding for between 150 and 167 open reading frames (1). ASFV is the causal agent of African swine fever (ASF), a reportable highly contagious disease of pigs and wild boar that represents a significant socio-economic threat for the pork industry worldwide (2).

A recent report of the World Organization for Animal Health (https://www.oie.int/app/uploads/2021/03/report-47-global-situation-asf.pdf) regarding the global situation of ASFV between 2016 and 2020 indicates that ASFV is endemic in most Sub-Saharan African countries and is causing outbreaks throughout Europe and Asia resulting in the loss of more than 6,000,000 domestic pigs, representing 82% of global losses to ASF during this time period.

In this context, the increased number of cases currently reported out of Africa are mostly attributed to the emergence of the Eurasian ASFV lineage (genotype II) (3), one of 23 ASFV genotypes (4). ASFV genotype II was first reported in the Republic of Georgia in 2007 and has subsequently spread to different countries in Asia and Europe (3, 5). Just recently (07/15/2021), The Friedrich-Loeffler-Institute reported the first cases of ASFV in domestic pigs in Germany (https://www.fli.de/en/news/animal-disease-situation/african-swine-fever), and (7/28/2021) The U.S. Department of Agriculture's (USDA) Foreign Animal Disease Diagnostic Laboratory confirmed the presence of ASFV in Dominican Republic, being this first report of this genotype in the Americas (https://www.aphis.usda.gov/aphis/newsroom/news/sa_by_date/sa-2021/asf-confirm).

Experimental infection of domestic pigs and wild boars with ASFV genotype II produced 100% mortality around 7 days post-infection (6–8), confirming the devastating effect of this virus to swine production. The latest epizootic has devastated swine industries across many countries in Europe and Asia, making development of an effective vaccine and a complementary diagnostic test that differentiates infected from vaccinated animals (DIVA) an international priority (9).

Currently, there is no commercial vaccine for ASFV, despite decades of work and multiple developmental strategies (3). Recently, experimental evaluation of three potential vaccine candidates obtained by deleting seven genes belonging to the MGF360 and MGF 505 families (ASFV-ΔMGF) (10), 9GL and UK (ASFV-G-Δ9GL/ΔUK) (11) or I177L (ASFV-ΔI177L) (12) demonstrated protection against challenge with the highly virulent ASFV Georgia 2007/1 strain. Recently, we published the adaptation of the recombinant ASFV-ΔI177L to grow in an established cell line (ASFV-ΔI177LΔLVR) and its potential to be used as a live attenuated vaccine (13). All four live attenuated vaccine candidates are in the process of being licensed in the U.S, with future possibility of commercialization. We developed genetic DIVA tests to support the use of these three vaccines in the field. For this purpose, three independent qPCR assays that detect the presence of MGF-360-12L, I177L and UK genes of ASFV were developed and validated. The results of this study are discussed in terms of the impact that these genetic DIVA marker tests may have in an outbreak situations as well as to support future experimental studies in pigs to evaluate the dynamic of the infection in vaccinated pigs challenged with the homologous virulent ASFV.

## Materials and Methods

### Viruses and Cells

Recombinant viruses ASFV-ΔMGF, ASFV-G-Δ9GL/ΔUK ASFV-ΔI177L and ASFV-ΔI177LΔLVR, all previously developed in our laboratory (10–13), as well as the parental virus ASFV Georgia 2007/1 strain (ASFV-G), a field isolate kindly provided by Nino Vepkhvadze from the Laboratory of the Ministry of Agriculture (LMA) in Tbilisi, Republic of Georgia were used to conduct this study.

Primary swine macrophage cell cultures were prepared from defibrinated blood as previously described (14).

### Primers and Probes Design

To detect target sequences of MGF-360-12L, I177L, and UK genes of ASFV, primers and probes were developed using the RealTime qPCR Assay tool from Integrated DNA Technologies (https://www.idtdna.com/scitools/Applications/RealTimePCR/).

**Figure 1:**
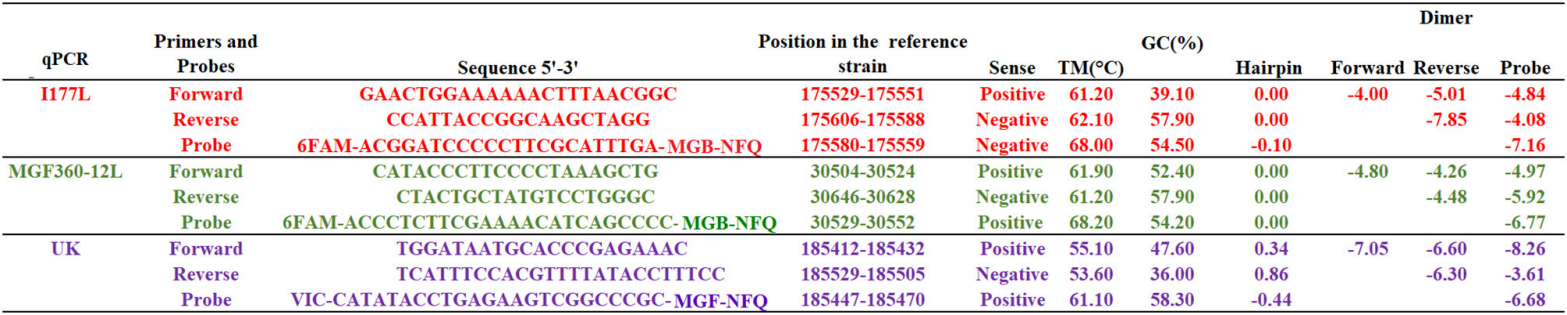
Sequences of primers and probes used in the qPCR reactions as well as multiple parameters calculated during these developments. Hairpin and dimer values are expressed in standard Gibbs free energy (ΔG kcal/mole). Designs were carried out using the RealTime qPCR Assay tool. Probes were labeled at 5′ with FAM (Fluorescein amidites) or VIC (Victoria) dyes and at 3′ with MGB-NFQ (Minor groove binder-non fluorescent quencher).

All primers and probes were designed based on the reference sequence of ASFV Georgia 2007/1 strain (GenBank data base NC_044959.2), considering the boundaries of the deletion of each gene as described in the publication of each vaccine candidate (10–12). The sequences of primers and probes are provided in Figure 1.

Additionally, to support the validation, specific primers and probes were designed for the detection of genes that code for fluorescent proteins present in each recombinant virus. For the detection of the mCherry gene, the sequence of the expression vector precB5R.1 (NCBI accession number: LC325569) was used as a reference sequence for the design of primers: forward, 5′-GCT TCT TGG CCT TGT AGG TG-3′, reverse, 5′-CAG AGG CTG AAG CTGAAG GA-3′, and probe, 5′-FAM-CGG CGG CCA CTA CGA CGC TG-MGB NFQ-3′.

The sequence of Gateway positive vector pENTR-gus (LC588893.1) was used for the design of primers and probe for the detection of the GUS gene encoding the protein beta-glucuronidase (β-GUS): forward, 5′-TCT ACT TTA CTG GCT TTG GTC G-3′, reverse, 5′-CGT A AG GGT AAT GCG AGG TAC, and probe, 5′-FAM-AGG ATT CGA TAA CGT GCT GAT GGT GC-MG B NFQ-3′.

### Standard Plasmid Control

A standard plasmid (pCloneEZ-NRS-Blunt-Amp) containing sequences of targeted regions of primers and probes from all designs was developed to support the validation of real-time PCRs. This plasmid was developed by Epoch Life Sciences, Missouri City, TX, USA.

### DNA Extraction

DNA extraction was conducted using a KingFisher automated extraction and purification system (ThermoFisher Scientific), using the MagMAX™ Pathogen RNA/DNA kit following the manufacturer instructions for 200 μl of sample.

### Real-Time PCR Performance

Real-time PCR assays were performed using an Applied Biosystems™ 7500 Real-time PCR system, using the TaqMan™ Universal PCR Master Mix (Applied Biosystems Catalog No. 4305719). Briefly, master mix was prepared in a final volume of 25 μl as follows: Universal mix 12.5 μl, primer forward (50 μM) 0.1 μl, primer reverse (50 μM) 0.1 μl, probe (10 μM) 0.25 μl, water 7.05 μl, and DNA sample 5 μl. Amplification conditions were as follows: Uracil N-glycosylase enzyme activation at 50°C for 2 min, polymerase activation at 95°C for 10 min; PCR of 40 cycles of 95°C for 15 s and 60°C for 1 min. Based on the validation, qPCR amplification of the I177L and UK targets was reduced to 37 cycles, and analysis used a manual threshold set at 0.1, with exception of the I177L target that utilized a 0.2 threshold.

As a gold standard to evaluate the performance of qPCRs designed in this study, we used the validated p72 qPCR, a reference test for the diagnosis of ASFV (15).

### *In silico* Primer and Probe Evaluation

To evaluate the potential of the qPCRs designed in this study to detect all ASFV genotypes, different primers and probes were assessed using the BLASTN tool, version 2.1.12.0 (16).

The results of this analysis were visualized in a phylogenetic tree. Full-length genomes that had 100% coverage of target areas were downloaded from GenBank. Sequence alignments were conducted using CLC Genomics Workbench, using a slow algorithm (very accurate) based on the progressive alignment method (17). MEGA X was used to construct the phylogenetic tree, using the neighbor-joining maximum likelihood method, with a bootstrap of 1,000 replicates (18).

### Amplification Efficiency (ε) and Analytical Sensitivity

To calculate the amplification efficiency (ε) of each qPCR, defined as the consistent increase in amplicon per cycle (19), 10-fold serial dilutions of the standard plasmid were produced using nuclease-free water. Average C_T_ values of each dilution were used to determine the amplification efficiency using the following equation:


$$\begin{array}{l} \varepsilon =100\;\times \;\left(10^{-1/\text{slope}}-1\right) \end{array}$$

Also, amplification efficiency was expressed as linearity (R^2^) (20).

Analytical sensitivity, defined as the smallest amount of the target template in the sample that can precisely be measured by qPCR (21), was calculated using the 10-fold serial dilutions of the standard plasmid, with the nucleic acid concentration previously calculated using the copy number calculator for real-time PCR (http://www.scienceprimer.com/copy-number-calculator-for-realtime-pcr). Values were calculated at the last dilution where different tests got the limit of detection, being 6/6 replicates detected. Final values for each test were expressed as target copy numbers.

Two additional experiments were performed to evaluate the analytical sensitivity of qPCR. To evaluate the analytical sensitivity of each test expressed as hemoadsorbing doses 50% per milliliter (HAD/_50_ doses/mL), multiple 10-fold dilutions were prepared from a viral stock of ASFV-G with a known titer of 1 × 10^8^ HAD/_50_ doses/mL. DNA from each dilution was extracted as previously described and qPCRs were performed in six replicates. The final dilution where 6/6 replicates produced C_T_ values was used to determine the limit of detection.

To assess the ability of each assay to detect low concentrations of ASFV-G in the presence of high concentrations of recombinant viruses, 10-fold dilutions made from a viral stock of ASFV-G were mixed with constant concentrations of different vaccine candidate stocks. DNA extractions and PCR reactions were performed using multiple mixes. Also, to evaluate how virus isolation can improve the sensitivity of the developed real-time PCRs, different mixes were used to infect primary swine macrophages, using plates containing 1 × 10^7^ cells per well. Based on the number of cells per well, the multiplicity of infection (MOI) for each recombinant virus in the mix was calculated as follows; ASFV-ΔI177L = 1, ASFV-G-Δ9GL/ΔUK = 0.003, and ASFV-ΔMGF = 0.01, while for ASFV-G the MOIs ranged between 0.01 and 0.000001. In all cases the initial concentration of the recombinant virus in the mix was determined based on the concentration of the original stock.

Briefly, cells were infected with 1 mL of the virus mixture, after 1 h of adsorption at 37°C the inoculum was removed, and cells were rinsed twice with PBS and then incubated at 37°C for 24 h. Finally, DNA was extracted, and qPCR reactions were performed and compared with the ones using the original mixes.

### Diagnostic Specificity

To calculate the diagnostic specificity, defined as the percentage of pigs that are not infected by ASFV and are identified by qPCR as negative for that condition (21), a total of 153 blood samples were evaluated. For this, two different sample sources were used. A total of 108 blood samples came from naïve pigs used in multiple previous ASFV experiments at PIADC (8–10; 19–23; (13)). An additional 45 blood samples were obtained from an unpublished experiment at PIADC that assessed the safety of different ASFV vaccine candidates. In this context, samples were collected from groups of three pigs inoculated with each of the vaccine candidates at 0, 7, 14, 21, and 49-days post inoculation (*n* = 15 samples per group).

Final values were expressed as a false positive rate using the following equation (20): False positive rate = (100 × number of misclassified known negative samples)/ (total number of negative samples).

### Diagnostic Sensitivity

To calculate the diagnostic sensitivity, defined as the percentage of pigs that are infected by ASFV and are correctly identified as positive for the presence of this virus by qPCR (21), 30 blood samples collected from pigs experimentally infected by intramuscular inoculation with ASFV-G (~1 × 10^2^ HAD/_50_ doses) and collected between 4 (*n* = 15) and 7 (*n* = 15) days post-challenge were used to evaluate the different qPCR tests (7, 8, 10–13, 22–24).

The capability of different qPCRs to detect minimal quantities of the desired DNA target was evaluated using serial 10-fold dilutions of the standard plasmid and from a virus stock with a known titer of ASFV Georgia strain. The averages of six independent repetitions were used to determine the limit of detection (LOD) of each of the developed real-time PCRs. LOD was expressed as DNA copy number and HAD_50_ doses.

Final values were expressed as a false negative rate using the following equation (20): False negative rate = (100 × number of misclassified known positive samples)/ (total number of positive samples).

### Virus Titrations

The virus titer from blood samples of viremic pigs was determined using primary swine macrophage cell cultures in 96-well plates, using hemadsorption (HA) as evidence of the presence of ASFV. After 7 days of incubation at 37°C, the Reed and Muench method was applied to determine the final virus titers (25).

## Results and Discussion

The prevention and control of ASFV is a major challenge for the global pork industry. In this context, during the last decade our research has been focused on the identification of essential virulence genes of ASFV. This research has led to the development of three promising vaccine candidates to promote the control of the Eurasian strain of ASFV (10–13). Herein, we present the development of three independent qPCRs to be used as complementary genetic DIVA tests, supporting the use of our vaccines in the field. The use of genetic DIVA tests has been applied to other swine diseases like classical swine fever, where this approach has been successfully used to differentiate animals vaccinated with the C-strain virus from animals infected with field strains (26).

### Real-Time PCR Design and *in silico* Evaluation

Using the sequence of the ASFV Georgia 2007/1 strain, we focused on the development of three independent qPCRs to target genes MGF360-12L, UK and I177L; these three genes were independently deleted to develop vaccine candidates ASFV-ΔMGF, ASFV-G-Δ9GL/ΔUK and ASFV-ΔI177L, respectively. Results obtained using the RealTime qPCR Assay tool are presented in Figure 1. Overall, *in silico* evaluation of different primers and probes reveled that all oligonucleotides had values of standard Gibbs free energy (ΔG kcal/mole) higher than −9 kcal/mol, a desired condition that may prevent excessive formation of hairpins and dimers that can interfere with amplification conditions (Figure 1).

**Figure 2:**
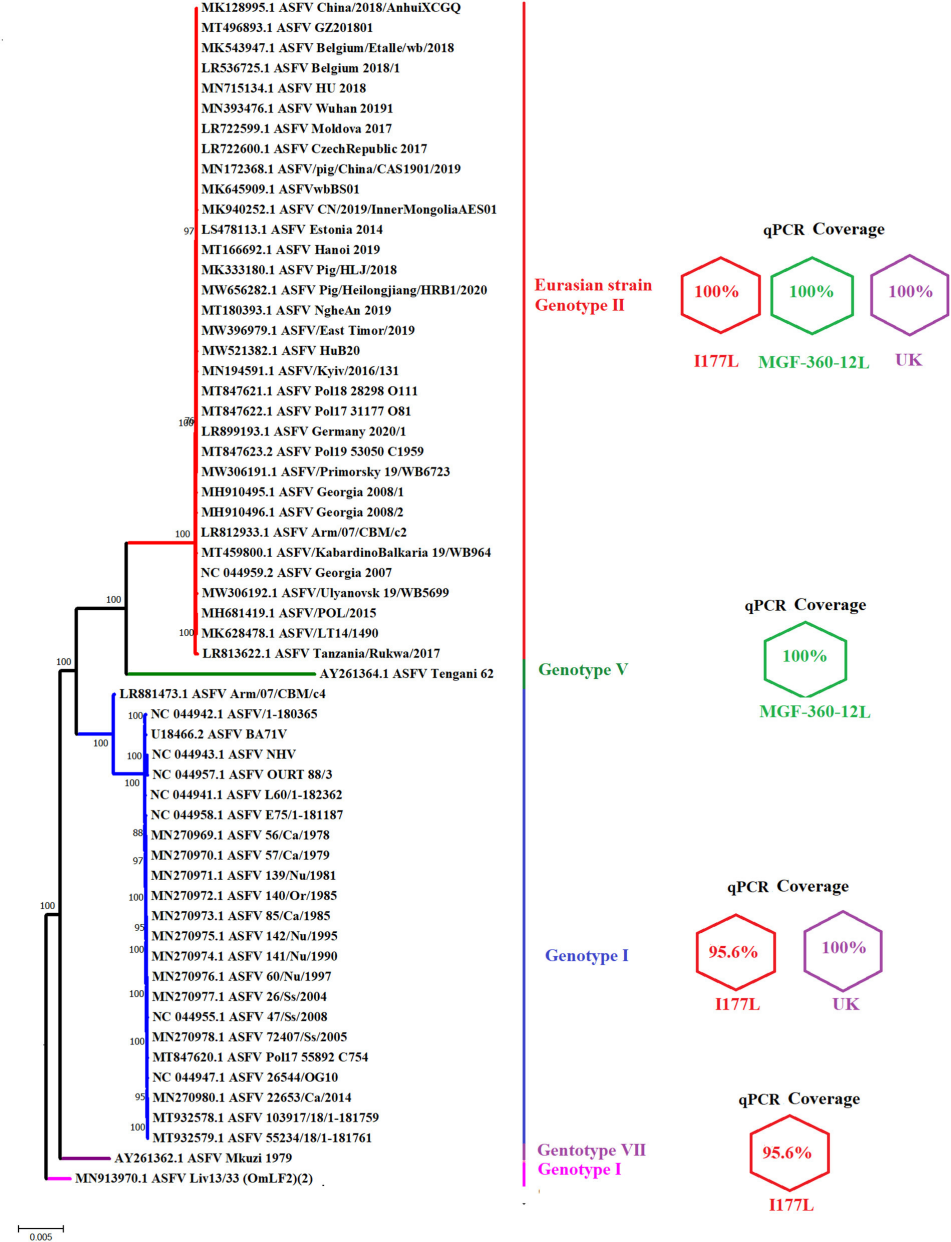
Graphic representation of the genetic coverage of different qPCR tests. The figure shows a phylogenetic analysis reconstructed by neighbor-joining using full-length sequences of multiple ASFV strains which may be potentially detected by different qPCRs designed in this study. Next to each clade representing multiple genotypes are expressed the identity of different primers and probes included in each qPCR. Values of 100% indicate no differences between viral sequences and different oligonucleotides. A value of 95.65%, for one I177L real-time PCR primer, is due to the presence of one mismatch between the forward primer and sequences of different genotypes. Numbers along the branches represent the bootstrap support values.

We then assessed the genetic coverage of the different real-time PCRs designed in this study. Primers and probes were evaluated using the software BLASTN. As expected, all oligonucleotides shared 100% nucleotide identity with the viral sequence of the ASFV Eurasian strain (genotype II). This was consistent with the results of our phylogenetic analysis that demonstrated high nucleotide conservation of the Eurasian strain after more than 12 years of circulation, appearing in the tree as a highly conserved monophyletic lineage (Figure 2). This result supported previous studies using the B646L gene as a genetic marker for the phylogenetic analysis (27, 28), suggesting that the genetic stability of this strain may favor the use of genetic DIVA tests as part of a control strategy.

Although the main goal of our study was to design different qPCRs to efficiently detect the Eurasian strain of ASFV (genotype II), our analysis would also demonstrate the ability to detect additional genotypes of ASFV with the developed assays. It would include the ability of MGF360-12L and UK designs to match 100% with viral strains associated with genotypes V and I, respectively (Figure 2). The I177L design appeared to detect strains associated with genotypes I and VII. However, while the I177L reverse primer and the probe were 100% identical, the forward primer was 95.6% identical due to a single mismatch.

Interestingly, the ASFV LIV 13/33 isolate, one of the strains potentially covered by the I177L design and classified as genotype I based on the B646L gene (29), was genetically distant from multiple strains of genotype I viruses using full-length sequences, suggesting the potential ability of the qPCR I177L to detect viral strains other than I, II and VII genotypes. Also, considering the high bootstrap values that support our analysis, our results agree with previous studies (29, 30) that suggest potential differences in the genotype classification of ASFV strains, dependent on selection of gene-specific or full-length genome sequence used for phylogenetic analysis.

### Amplification Efficiency and Analytical Sensitivity Determinations

Part of the *in vitro* validation of the qPCRs was the calculation of their amplification efficiency and analytical sensitivity parameters. For this purpose, serial 10-fold dilutions of a standard plasmid containing all different PCR targets was used for the determinations. As a gold standard for this validation, we included the previously validated qPCR for the detection of the B646L gene (p72 protein), a standard assay for field diagnosis of ASFV (15).

**Figure 3:**
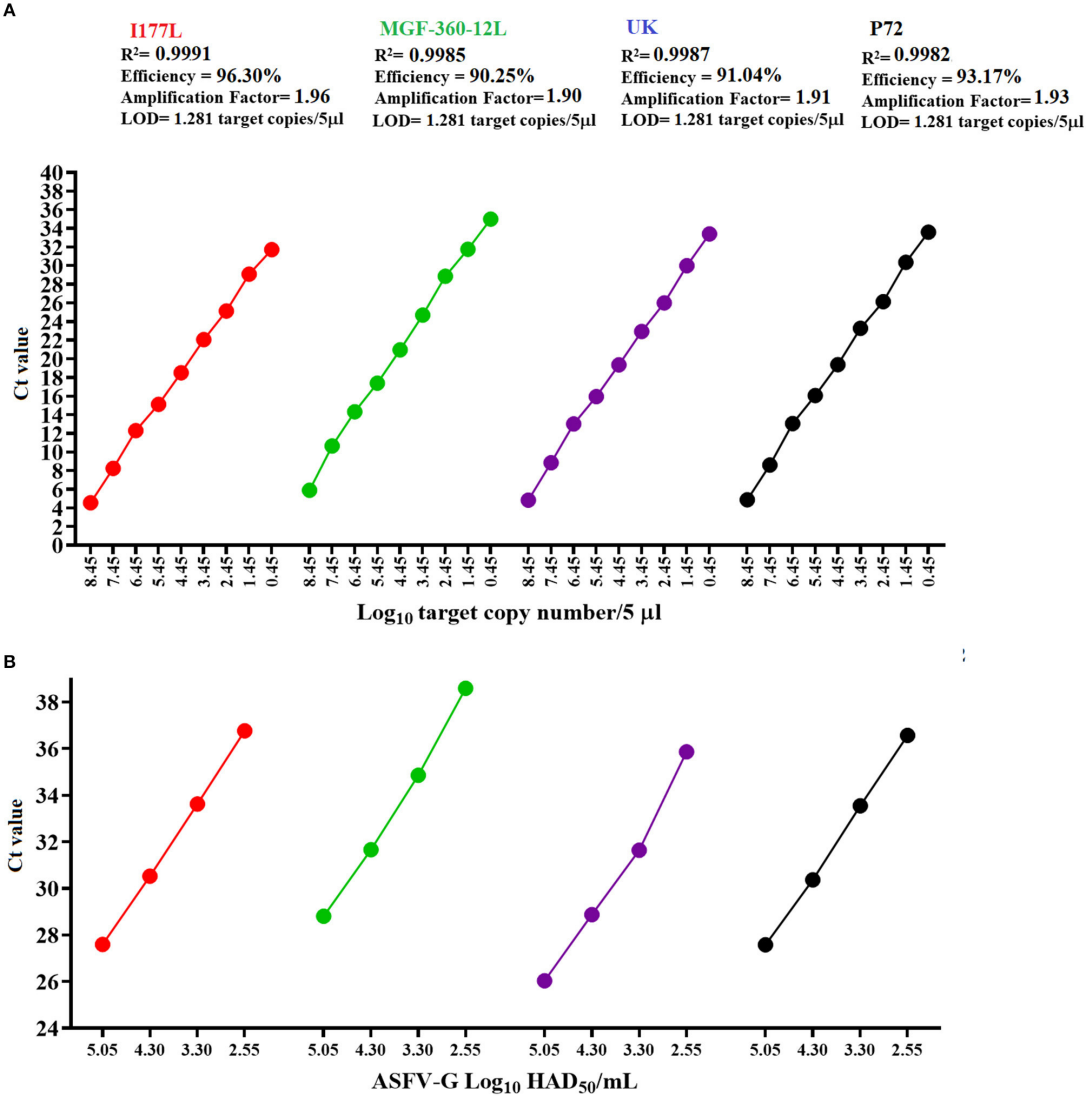
Amplification efficiency and analytical sensitivity determinations. **(A)** Ten-fold dilutions of a standard plasmid representing variable amounts of all gene targets were used to calculate the amplification efficiency and analytical sensitivity of multiple qPCRs. **(B)** Ten-fold dilutions of a stock of ASFV-G with a known titer was used to establish parameters of analytical sensitivity in terms of the capability of different qPCRs to detect minimal amounts of infectious ASFV. Amount of infectious virus is expressed as HAD_50_/mL units. In all cases results represent the average value of six replicates.

Overall, all three real-time PCRs designed in this study had comparable values of amplification efficiency when compared with the p72 qPCR (Figure 3A). These results agree with the previously proposed accepted standard values for amplification efficiency based on the determination of an amplification factor (between 80 and 120%), expressed as linearity (R^2^), where the acceptable values for each target should be ≥0.98 (20).

Analytical sensitivity of all three qPCRs, like the p72 qPCR, demonstrated the capability to detect 1.28 copies of each of the gene targets (Figure 3A). The limit of detection for all assays was achieved with C_T_ values < 36, increasing the chances to obtain consistent levels of the repeatability during the performance of these assays (31). Also, when compared to other designs, our results were similar to a previously reported qPCR developed to detect the MGF505 gene of ASFV (3 copies of the target gene) (31), supporting the robustness of our assays to detect minimal amounts of ASFV from pig samples collected in the field.

Furthermore, when analytical sensitivity was assessed in terms of the ability of different designs to detect minimal amounts of infectious virus quantified as HAD_50_/mL, the detection was consistent with the results showed by the standard diagnostic p72 qPCR (2.55 HAD_50_/mL) (Figure 3B). Interestingly, all these calculations were consistent with the values obtained in the original validation of the p72 real-time PCR (15), supporting the reliability of our results.

### Assessing the Presence of ASFV-G in a Combined Infection With Different Recombinant Viruses

We evaluated the performance of multiple qPCRs to detect different concentrations of ASFV-G in the presence of constant levels of the three different recombinant viruses. The presence of both viruses circulating in the blood of vaccinated and infected pigs is a possible field scenario, since experimental evidence has shown the absence of sterile immunity in a proportion of pigs vaccinated with ASFV-ΔMGF or ASFV-G-Δ9GL/ΔUK (10, 11).

**Figure 4:**
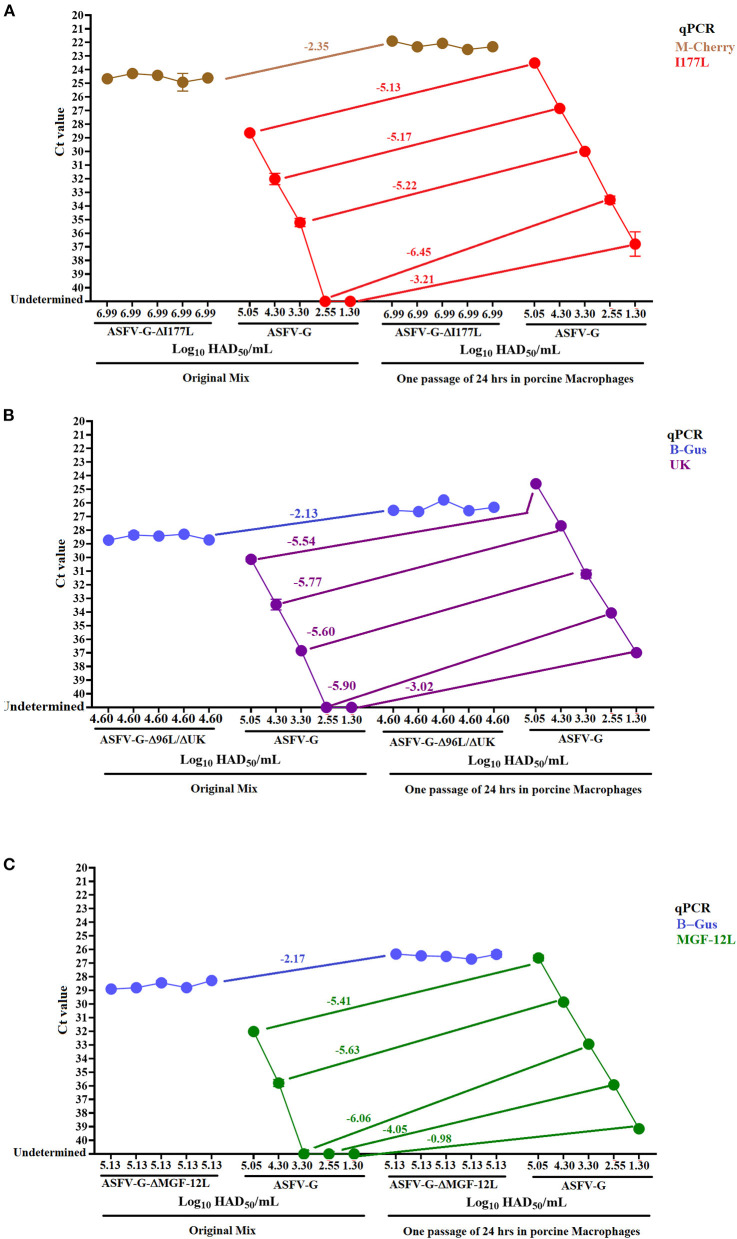
Detection of ASFV-G in the presence of recombinant viruses. The sensitivity of different real-time PCRs was evaluated using mixes obtained from combined infections using constant concentrations of recombinant viruses and variable amount of ASFV-G **(A)** ASFV-ΔI177L, **(B)** ASFV-G-Δ9GL/ΔUK, and **(C)** ASFV-ΔMGF. This evaluation was carried out in both the original virus mixes and after being passed once in porcine macrophage. The detection of recombinant viruses was conducted using specific qPCRs to detect the gene encoding the markers M-cherry (ASFV-ΔI177L) and β-Gus (ASFV-G-Δ9GL/ΔUK and ASFV-ΔMGF).

Interestingly, in the presence of the recombinant viruses the qPCR detection of ASFV-G decreased its levels of analytical sensitivity by ten- (I177L and UK) (Figures 4A,B) or 100-fold (MGF-360-12L) (Figure 4C). However, after one 24-h passage in cell culture of porcine macrophages, all qPCRs restored and improved their levels of analytical sensitivity to detect samples with original titers as low as 10^1.30^ HAD_50_/mL. After this passage in porcine macrophages, there was a reduction in average C_T_ values of all three qPCRs used to detect ASFV-G when compared with the C_T_ values obtained by qPCRs targeting the florescent markers in the recombinant viruses (Figure 4).

It is possible that the overall loss of analytical sensitivity of all qPCRs may be impacted by the extraction method used in this study, where the higher concentrations of recombinant virus present in all mixes might have favored the attachment of the DNA from this virus to the magnetic beads. This possibility highlights the necessity to explore alternative extraction methods to improve the performance of these tests (32). Regarding the increased loss of analytical sensibility seen from the MGF-360-12L design, it may be explained by the lowest level of amplification efficiency showed by this test in comparison with the other qPCRs designed in this study (Figure 3).

Therefore, the combined use of virus isolation and qPCR may be an alternative to consider in order to improve the performance of genetic DIVA tests to rule out the presence of ASFV in pigs vaccinated with recombinant viruses in the field. Experimental evidence indicates that in pigs vaccinated with ASFV-ΔMGF and ASFV-G-Δ9GL/ΔUK the infection with ASFV-G were asymptomatic, so that low levels of viremia are expected (10, 11). ASFV isolation typically requires the use of primary cell cultures of swine macrophages, however the recently identified adapted cell line MA-104 may be an alternative to be considered for this purpose particularly when primary cell cultures are not available (33).

### Diagnostic Sensitivity

**Figure 5:**
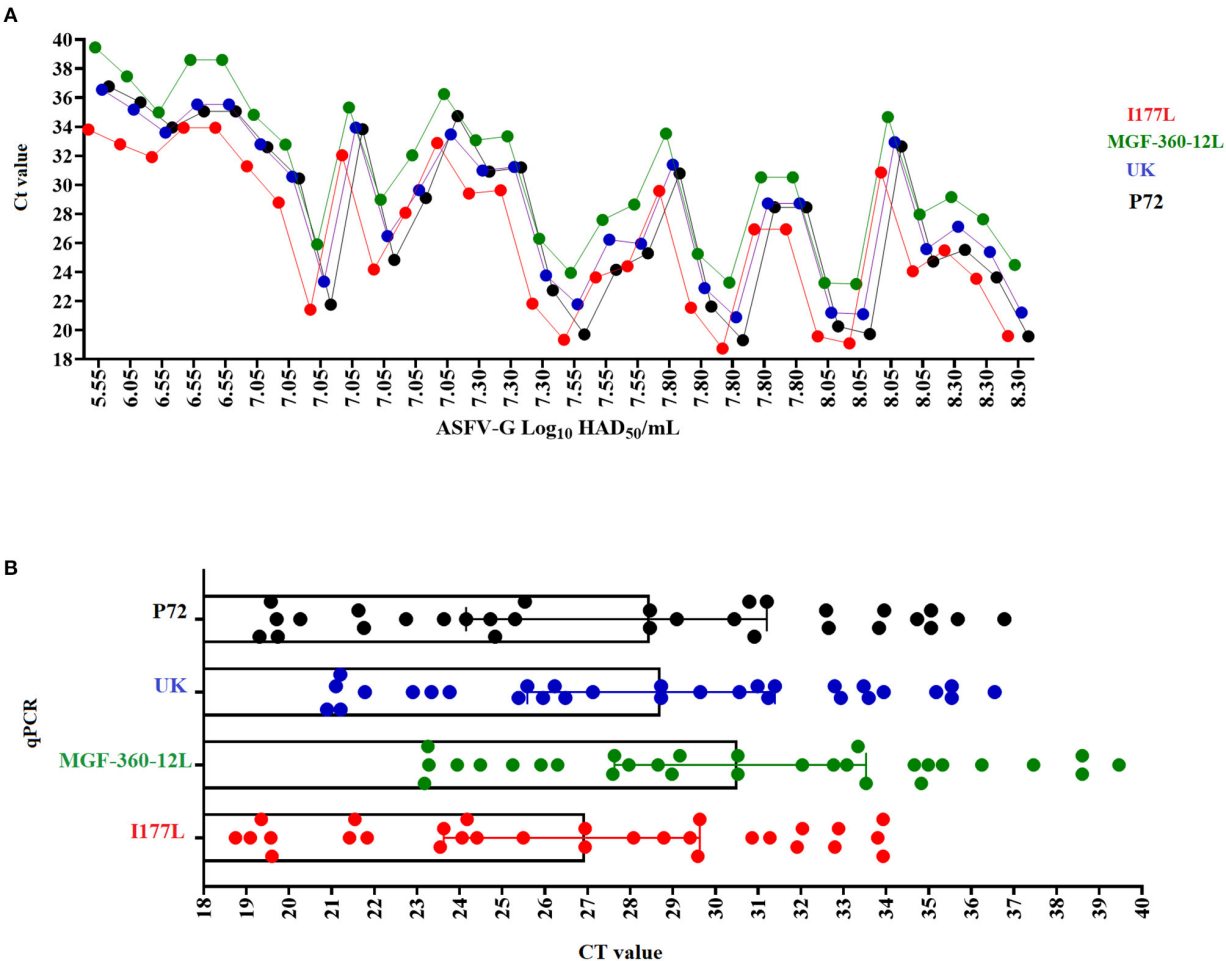
Diagnostic sensitivity. of the different qPCRs assessed using a set of blood samples with different viral titers (expressed in HAD50/ml) collected from pigs experimentally infected with ASFV-G **(A)**. Comparative average C_T_ values among the different qPCRs in the detection of viremic blood samples obtained from pigs infected with ASFV-G **(B)**.

The diagnostic sensitivity of different qPCRs was evaluated using a set of blood samples from viremic pigs infected with an average of 10^2^ HAD/_50_ doses of ASFV-G and collected between days 4 and 7 post-infection. In general, all qPCRs were able to detect 100% of the samples tested for this validation, producing a false positive rate = 0% (Figure 5A). The average C_T_ values for the detection of all samples were consistent with the levels of amplification efficiency calculated for each qPCR, with the lowest values associated with the I177L test and the highest with the MGF-360-12L test (Figure 5B). Interestingly, we found an absence of a positive linear correlation between the viral titer and the C_T_ values produced by all qPCRs, including p72, which may be explained by the presence of PCR inhibitors in the samples decreasing the diagnostic sensitivity of these tests. In blood the presence of inhibitors may be associated with substances like antibodies (IgG), hemoglobin, lactoferrin, heparin, hormones, and some antiviral agents (34, 35).

In this context, the use of alternative sample types, like nasal, oral swabs, and the collection of oral fluids may represent a good alternative to maintain optimal levels of diagnostic sensitivity in these tests (15, 36). Further studies will involved the evaluation of different types of clinical samples, as oral, rectal and nasal swabs.

In light of these results, we can state that despite the apparent loss of analytical sensitivity produced by blood inhibitors, the high levels of viremia that are expected in domestic or wild pigs during clinical infection with the highly virulent Eurasian strain of ASFV (6, 24) may help to ensure the proper levels of diagnostic sensitivity of these tests when used in the field. However, it is important to consider the recent reports regarding the circulation of low virulent genotype II ASFV strains (37, 38), a situation that may affect the diagnostic sensitivity of these tests considering that blood is used as a primary sample for the performance of these tests. Interestingly, experimental infections comparing the pathogenesis among ASFV isolates (genotype II) of disparate levels of virulence have shown that blood can be isolated from pigs infected with strains with low and moderate levels of virulence as late as 19- and 44-days post-infection respectively, thus supporting the use of blood as a valuable sample for the detection of ASFV genotype II (37).

Furthermore, consistent with the original publications [8–10; (13)], the evaluation of blood collected from pigs (*n* = 5 per group) vaccinated and then challenged 21 days later, the qPCRs designed herein did not produce positive results in pigs vaccinated with ASFV-ΔI177L, a fact consistent with the previously described ability of this vaccine to produce sterile immunity in vaccinated pigs. Conversely, one out of five animals (20%) vaccinated with ASFV-ΔMGF had a positive result by qPCR, while 3 out of 5 pigs (60%) vaccinated with ASFV-G-Δ9GL/ΔUK had positive results.

### Diagnostic Specificity

**Figure 6:**
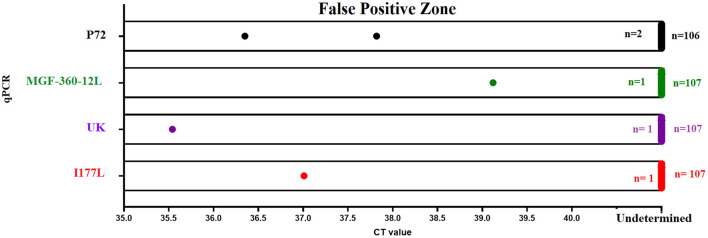
Diagnostic specificity. The diagnostic specificity of different qPCRs was assessed using a set of 108 blood samples collected from naïve pigs. Results from different tests represent the detection of nonspecific reactions after 40 (p72 and MGF-360-12L) or 37 (I177L and UK) cycles of amplification.

Finally, we assessed the diagnostic specificity of the designed real-time PCR tests. We evaluated a total of 108 negative blood samples collected from naïve pigs. Similar results were seen between p72 and all qPCRs developed in this study. The overall false positive rate was estimated to be less than 1%, due to 1/108 positive result (Figure 6). The amplification profile of this sample was characterized by the amplification of just one of the two replicates evaluated; considering that none of the samples were positive by two different tests, we determined this was a nonspecific detection.

It is important to mention that in the case of real-time PCRs I177L and UK, we noticed that a small percentage of samples (5%) reported a nonspecific amplification in one of the 2 replicates, with C_T_ values >38. Interestingly, as mentioned above, none of these blood samples had a positive amplification in two different real-time PCRs. In this context and based on the parameters of analytical and diagnostic sensitivity displayed by the I177L and UK tests, amplification protocol for these tests was set at 37 amplification cycles instead of 40, producing in this way the presence of just one sample showing a nonspecific amplification in one out of the two repetitions (Figure 6).

At this point, we cannot rule out the possibility that the increased number of nonspecific reactions recorded using the I177L and UK qPCRs might have been the result of dimer formation between different primers, so that alternative primer technology like the use of cooperative primers may be explored in future studies to improve this condition (39). Furthermore, negative results were recorded when multiple qPCR's were performed in the presence of other viral swine diseases like classical swine fever virus (CSFV), vesicular stomatitis virus (VSV) and foot and mouth disease virus (FMDV).

**Figure 7:**
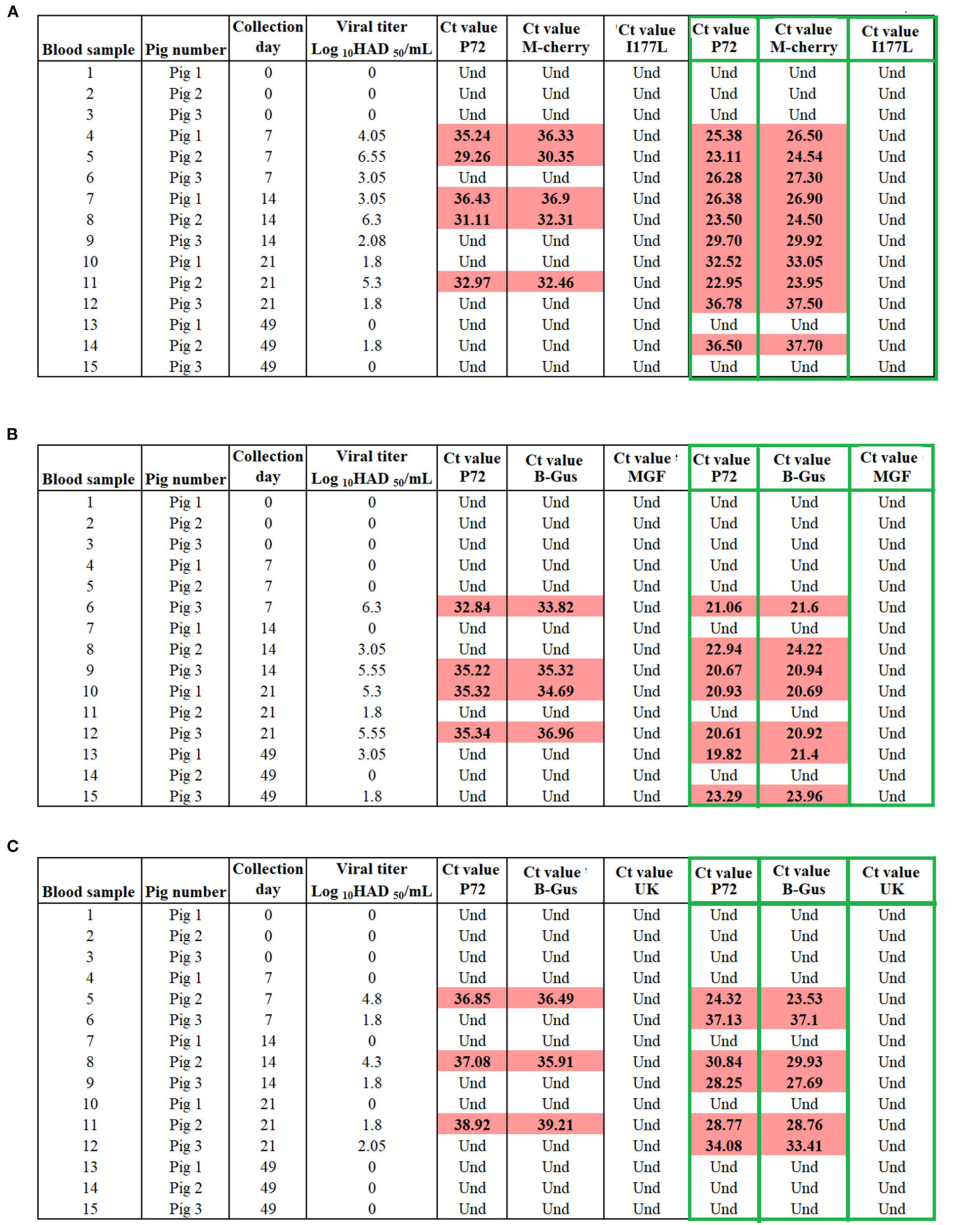
Evaluation the capability of qPCRs to differentiate vaccinated pigs with **(A)** ASFV-ΔI177L, **(B)** ASFV-G-Δ9GL/ΔUK, and **(C)** ASFV-ΔMGF from those infected with ASFV-G. Blood samples collected from pigs at different time points of vaccination were tested before and after (green boxes) one passage of 72 h in swine macrophage cell cultures. M-cherry and β-Gus q PCRs were used for the detection of the recombinant viruses, while p72 assay was used as a marker for the presence of ASFV.

Alternatively, to estimate the diagnostic specificity of different designs in samples containing variable concentrations of recombinant viruses, we evaluated blood samples collected at different time points post-vaccination from groups of viremic pigs (*n* = 3 animals per group) inoculated with different recombinant viruses. Overall, positive results were obtained by p72, M-cherry and β-Gus qPCRs in blood collected from all groups at different time points, denoting the presence of different recombinant viruses in the blood of vaccinated pigs. The number of positive results increased in all groups of pigs after passing these samples once in cell cultures of porcine macrophages (Figure 7). Negative results were found in all blood samples when evaluated by I177L, MGF-360-12L, and UK qPCRs, confirming the absence of ASFV-G in the samples, confirming the ability of these tests to differentiate infected and vaccinated pigs.

In conclusion, we present the design of three independent genetic DIVA tests for use in the field in the presence of recombinant vaccines ASFV-ΔMGF, ASFV-G-Δ9GL/ΔUK, ASFV-ΔI177L and ASFV-DI177LDLVR. Future studies are being planned to conduct a full validation under field conditions and confirm the accuracy of the validation parameters established here. The qPCR DIVA tests developed here are a promising option to support the control and eradication of the ASFV-G strain during a potential vaccination program. In addition to the vaccine strains tested here, these qPCR tests would also be appropriate for experimental vaccines developed by other groups using Chinese strains of ASFV. The qPCR DIVA test for UK could identify ASFV-SY18-ΔCD2v/UK (40), the MGF qPCR DIVA test could be used to detect HLJ/18-6GD (41) and HLJ/18-7GD (41) since in all of these experimental vaccine candidates the viral sequence is 100% homologous to the primer sets tested here in this study making the qPCR DIVA tests potentially useful in areas where a potential vaccine program may use one of the experimental vaccines.

## Data Availability Statement

The original contributions presented in the study are included in the article/supplementary material, further inquiries can be directed to the corresponding authors.

## Ethics Statement

The animal study was reviewed and approved by Animal experiments were performed under biosafety level 3 conditions in the animal facilities at Plum Island Animal Disease Center, following a strict protocol approved by the Institutional Animal Care and Use Committee (225.01-16-R approved on 09-07-16).

## Author Contributions

LV-S, MB, and DG conceived and designed the experiments and analyzed the data. LV-S, ER-M, AR, SP, EV, and NE performed the experiments. LV-S, MB, DG, ER-M, AR, SP, EV, and NE wrote the manuscript. All authors contributed to the article and approved the submitted version.

## Funding

This work was performed under USDA Research Service CRIS Project No. 8064-32000-060-00D.

## Conflict of Interest

DG and MB have licensed the vaccine platforms mentioned in this study to commercial partners. The remaining authors declare that the research was conducted in the absence of any commercial or financial relationships that could be construed as a potential conflict of interest.

## Publisher's Note

All claims expressed in this article are solely those of the authors and do not necessarily represent those of their affiliated organizations, or those of the publisher, the editors and the reviewers. Any product that may be evaluated in this article, or claim that may be made by its manufacturer, is not guaranteed or endorsed by the publisher.
